# Urea-Assisted Synthesis and Characterization of Saponite with Different Octahedral (Mg, Zn, Ni, Co) and Tetrahedral Metals (Al, Ga, B), a Review

**DOI:** 10.3390/life10090168

**Published:** 2020-08-28

**Authors:** Concepcion P. Ponce, J. Theo Kloprogge

**Affiliations:** 1 Department of Chemistry, College of Arts and Sciences, University of the Philippines, Miag-ao, Iloilo 5023, Philippines; cpponce@upv.edu.ph; 2 School of Earth and Environmental Sciences, The University of Queensland, Brisbane, Queensland 4072, Australia

**Keywords:** clay minerals, heterogeneous catalysis, saponite, synthesis, urea

## Abstract

Clay minerals surfaces potentially play a role in prebiotic synthesis through adsorption of organic monomers that give rise to highly concentrated systems; facilitate condensation and polymerization reactions, protection of early biomolecules from hydrolysis and photolysis, and surface-templating for specific adsorption and synthesis of organic molecules. This review presents processes of clay formation using saponite as a model clay mineral, since it has been shown to catalyze organic reactions, is easy to synthesize in large and pure form, and has tunable properties. In particular, a method involving urea is presented as a reasonable analog of natural processes. The method involves a two-step process: (1) formation of the precursor aluminosilicate gel and (2) hydrolysis of a divalent metal (Mg, Ni, Co, and Zn) by the slow release of ammonia from urea decomposition. The aluminosilicate gels in the first step forms a 4-fold-coordinated Al^3+^ similar to what is found in nature such as in volcanic glass. The use of urea, a compound figuring in many prebiotic model reactions, circumvents the formation of undesirable brucite, Mg(OH)_2_, in the final product, by slowly releasing ammonia thereby controlling the hydrolysis of magnesium. In addition, the substitution of B and Ga for Si and Al in saponite is also described. The saponite products from this urea-assisted synthesis were tested as catalysts for several organic reactions, including Friedel–Crafts alkylation, cracking, and isomerization reactions.

## 1. Introduction

Clay minerals are a group of minerals that occur as colloidal crystals in sediments and soils. They consist mostly of hydrated aluminum phyllosilicates, which may contain variable amounts of iron, magnesium, alkali metals, alkaline earths, and other cations found on or near some planetary surfaces. They are abundant and widely distributed in nature showing a wide variety of structures, compositions, and properties. Clay minerals have been posited to figure prominently in the origin of life. Bernal was the first to point to the potential role of clay minerals in abiogenesis [1]. He suggested that surfaces of clay minerals are the likely location where primitive biomolecules that occurred in the “primordial soup” could concentrate and be available for further reactions. Cairns-Smith and Hartman forwarded the clay hypothesis and postulated that complex organic molecules arose gradually on pre-existing, non-organic replication surfaces of silicate crystals in solution [2]. A few others have supported the importance of clays in the synthesis of simple and organic molecules that are the building blocks of life. Among them are Balogh and Laszlo, who provided an overview of organic reactions catalyzed by clays, whether natural, activated and modified, including pillared clays or performed by clay-supported reagents [3]. Ferris and his group showed that ribonucleic acid (RNA) oligomers (RNAs are capable of both information storage and as templates to catalyze the synthesis of complementary molecules) can be synthesized on the surfaces of montmorillonite [4,5,6,7]. These montmorillonite surfaces were also shown to influence the regioselectivity of the RNA oligomers. [5]. The indication that clay surfaces played an important role came about because the environment of the early ocean would favor the following phenomena: (1) slow synthesis of the amino acids, nucleotides and other building blocks of life; (2) thermodynamically-hindered formation of proteins, RNA and DNA from these building blocks; and (3) more favored hydrolysis and photolysis of these complexes. Adsorption onto surfaces and their consequent interfacial interactions have been shown to overcome these obstacles. In fact, Huang and Ferris had developed a “one step,” no primer method for the synthesis of RNA oligomers in the presence of clays [8]. Negron-Mendoza et al. further espoused that the most geologically relevant solid surfaces to promote chemical reactions on the primitive Earth are clay minerals [9]. Such claims are based on observations that clay minerals are ubiquitous minerals on Earth and very likely appeared in the early steps of the formation of our planet; they have been possibly present at least 3.8 × 10^9^ years ago, as indicated in the analysis of the Isua sediments in Greenland [10]. 

The surfaces of clay minerals may have played a role in prebiotic synthesis through adsorption of monomers that give rise to highly concentrated systems, facilitate condensation and polymerization reactions, protection of early biomolecules from hydrolysis and photolysis, and surface-templating for specific adsorption and synthesis of organic molecules. Several clay minerals, such as smectite-type clays [11,12], including montmorillonite [5,6,13,14,15,16] and saponite [12,17], layered double hydroxides [18,19], etc., were experimentally shown to play these roles. While papers on the origin of life centered more on the role of clay minerals in prebiotic synthesis as seen in reviews by Ponnamperuma et al. [20], Ferris [21], and Brack [22], this review presents processes of clay formation using saponite as a model clay mineral since an understanding of how clay minerals are formed is key to understanding the roles played by these minerals in the origin of life. Small organic molecules present in hydrothermal fluids that permeated through the pores of early Earth’s crust could have assisted in the formation of clay minerals. This, in turn, could have formed clay microenvironments for adsorption and interaction with other organic molecules, which could have led to the formation of more complex organic molecules like DNA or RNA, proteins, etc.

The choice of saponite is based on studies that show Fe-rich saponite were able to promote and preserve precursors of biopolymers and this may have contributed to prebiotic chemistry on Earth and the deep biosphere [17]. Sueoka et al. studied the mineral-filled fractures of a basaltic rock core sample obtained during the Integral Ocean Drilling Project Expedition 329 and found that these minerals were rich in Mg-rich saponite and calcium carbonate [23]. The saponite-rich clay fraction in the core contains a much higher organic carbon than the bulk counterpart suggesting enough supply of energy and carbon sources for a saponite-hosted life [23]. Apart from the possible contributions of saponites to early life, another reason for the interest in the system is the observation that saponites are easy to synthesize and their structures can be functionalized and modulated to obtain a wide variety of interesting novel advanced layered materials for use in heterogeneous catalysis [17] or for protection of organic compounds from photolysis [24]. In addition to the choice of saponite as this article’s focus, urea, a compound easily formed by the hydrolysis of cyanide and is often used as model compound in classic prebiotic reactions, is also given emphasis. Urea is a very interesting molecule. Its structure and properties make it a potentially good starting material for the synthesis of nucleobases and related molecules [25,26]. It has been shown to promote phosphorylation reactions [25,26]. Moreover, it was able to catalyze formation of saponite clays in only 20 h under relatively mild conditions [27]. This review, thus, presents the (1) various methods of preparation of saponite clays giving emphasis to those syntheses where urea plays a significant role; (2) tools that follow and ascertain their formation from precursor materials, as well as probe their structural and property changes as they interact with their environment; and 3) thermal stability and catalytic properties of synthetic saponites.

## 2. Saponite Synthesis and Characterization 

### 2.1. Saponite Composition and Occurrence

**Figure 1:**
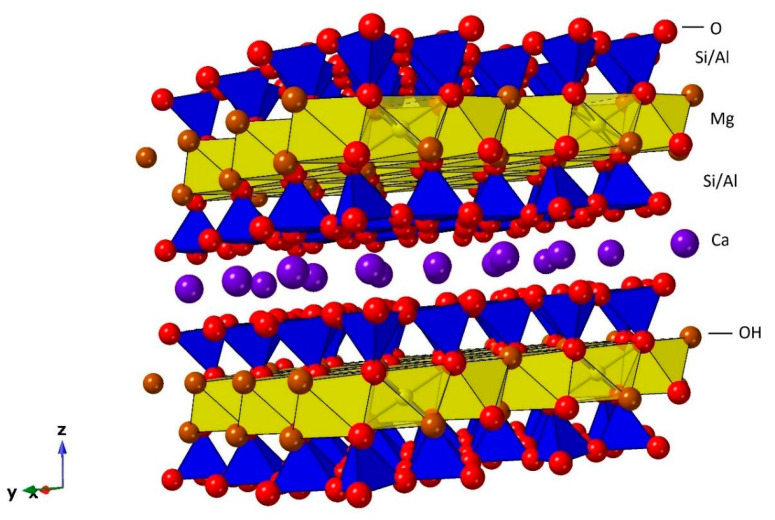
A schematic representation of saponites showing the major elements occupying the tetrahedral, octahedral sheets and interlayer spaces.

Saponite is a 2:1 type trioctahedral member of the smectite group of clay minerals with the theoretical chemical formula Ca_0.25_(Mg,Fe)_3_((Si,Al)_4_O_10_)(OH)_2_·n(H_2_O). Calcium can be substituted by sodium (Na^+^) and potassium (K^+^) cations. Mg^2+^ dominates in the octahedral sheets but with some Al^3+^, Fe^2+^, Fe^3+^, Li^+^, Mn^2+^, Ni^2+^, and Ti^4+^ substitutions occurring while Al^3+^ and Fe^3+^ for Si^4+^ substitutions occur in the tetrahedral sheets (Figure 1) [27]. Saponite is soft, massive, and plastic, and exists in hydrothermal veins; basalt vesicles; and fissures cutting calc-silicates, iron-rich skarns, amphibolites and serpentinites. The name is derived from the Latin sap, making allusion to its greasy and soapy feel and appearance. 

### 2.2. Preparation Methods

Several preparation methods of synthetic saponites with tunable composition and physico-chemical properties have been reported in the literature. These methods are grouped into sol-gel processes under mild temperatures and pressures; hydrothermal processes which are carried out at relatively higher temperatures and pressures; and microwave-assisted hydrothermal synthesis, which allows for synthesis to occur at a temperature lower than typical hydrothermal methods and at a much shorter timescale. Table 1 summarizes the starting materials and conditions for the synthesis of saponites. 

The hydrothermal methods are the most explored procedures for the synthesis of saponites. Kloprogge and his co-researchers presented several works on the hydrothermal synthesis of saponites [28,29,30,31,32]. Their works suggested that hydrothermal techniques are successful in obtaining pure products, due to the high hydrolysis rates of the octahedral cations precursors at high temperature, which favor nucleation and growth of saponite materials. Hydrothermal methods involve a gel with stoichiometric mixture of silica, Al and Mg salts and a source of interlayer cations (typically sodium or ammonium) which is treated under hydrothermal conditions at temperatures ranging from 150–450 °C under autogenous water pressures for a period of 5 min to as long as 3 months (Table 1).

**Table 1:** Saponite synthesis methods.

Author/s, Year	Starting Materials	Conditions	Product/Product Properties	Notes
Kloprogge et al., 1993 [28]	SiO_2_, Al[OCH(CH_3_)_2_}_3_, Mg(CH_3_COO)_2_.4H_2_O, NH_4_OH; gel composition: (NH_4_)_0.6_Mg_3_Al_0.6_Si_3_._4_O_10_(OH)_2_	Autoclave at 125 to 280 °C for 72 h	NH_4_-saponite—high crystallinity, low CEC, high Al content in interlayer spacing or in octahedral sites	uncontrolled intercalation of Al^3+^ in the interlayer; non-swelling saponites with low amounts of NH_4_^+;^ deceased catalytic activity
Kloprogge et al., 1994 [29]	modified from Kloprogge et al., 1993; solutions containing the desired cation were in the form of a hydroxide or fluoride salt; gel composition: (M)_0.6_Mg_3_Al_0.6_Si_3_._4_O_10_(OH)_2_; (M = Na^+^, K^+^, Rb^+^, Ca^2+^, Ba^2+^, or Ce^4+^)	Autoclave at 200 °C and autogenous water pressure for 72 h	Mg-saponite—low CEC, low stacking of saponite sheets; considerable octahedral aluminum substitution;	(1) the presence of interlayer Mg^2+^ and the absence of interlayer Al^3+^; (2) a much higher amount of octahedral AI; and (3) that synthesis experiments with fluorine do not result in incorporation of extensive F- into the saponite structure replacing hydroxyl groups, nor in the formation of sellaite, MgF_2_
Kawi and Yao, 1999 [33]	sodium silicate solution; NaOH/NaHCO_3_; chlorides of M^3+^ = Al^3+^, and M^2+^ = Mg^2+^, Ni^2+^; saponite of theoretical formula [MgxNi_6_-_x_](Si_7_Al)O_20_(OH)_4_	Autoclave at 285 °C for 48 h	saponite with varied Mg/Ni ratio	Mg and Ni were incorporated in octahedral sites; tested as catalyst for dehydration of IPA to propene and dehydrogenation to acetone
Vogels et al., 1997 [31]	modified from Kloprogge et al., 1993; stoichiometric powder mix of SiO_2_ and Mg(CH_3_COO)_2_⋅4H_2_O; NH_4_Cl or NH_4_F with the Al[OCH(CH_3_)_2_}_3_ dissolved in the aqueous ammonium solutions before mixing with the powder	Autoclave at 200 °C and autogenous water pressure; varying synthesis time	NH_4_-saponite	The crystallinity of synthetic ammonium-saponite depends strongly on synthesis time, ammonium concentration and initial constituents of the gel.
Higashi, Miki and Komarmeni, 2007 [34]	silicic acid (containing 81.4% SiO_2_), MnCO_3_, Al(NO_3_)_3_∙9H_2_O, NaOH solution	Autoclave at 100–250 °C under autogenous pressure for 72–168 h	Mn-saponite with Mn-carbonate impurities	Was not analyzed further due to poor crystallinity.
Carniatto et al., 2009 [35]	modified from Kloprogge et al., 1993; SiO_2_, Al[OCH(CH_3_)_2_}_3_, Mg(CH_3_COO)_2_.4H_2_O, NH_4_OH, vanadium(IV) oxide sulphate hydrate (VOSO_4_∙xH_2_O; tetraethyl orthosilicate (TEOS); acidified ethanol	Autoclave at 240 °C for 72 h	V-saponite, crystalline	Strong potential as additive for polymer materials with flame retardant properties.
Bisio et al., 2011 [36]	modified from Kloprogge et al., 1993; the reagents enumerated to form the gel in Kloprogge et al., 1993 were added with hexadecyltrimethylammonium bromide (CTABr)	Autoclave at 200 °C for 72 h	organo-saponite	CTA- ions are essentially confined in the interlayer space of the saponite.
Sychev and Prihod’ko, 1998 [37]	Na_2_SiO_3_; NaOH; M^3+^-nitrate where M = Al, Fe or Cr; aqueous M^2+^-nitrate and urea; Si/M^3+^ ratio is varied from 3.0–12	Sol-gel precipitation at 190 °C at 1 atm for 24 h	saponite like materials; poor crystallinity; high CEC	Possess acidic and basic/redox active sites which depends on the chemical composition of both tetrahedral and octahedral sheets of saponite.
Vogels et al., 2005 [27]	stoichiometric mixture containing Si/Al^3+^ gel from Na_2_SiO_3_ solution and Al(NO_3_)_3_∙9H_2_O; NaOH solution, M^2+^-nitrate (M^2+^ = Mg^2+^, Zn^2+^, Ni^2+^, Co^2+^, or Cu^2+^), urea, and water	Sol-gel co-precipitation at 90 °C for 20 h	M^2+^-saponites where (M^2+^ = Mg^2+^, Zn^2+^, Ni^2+^, Co^2+^, or Cu^2+^)	Thermal stability increases in order Zn^2+^, Co^2+^, Mg^2+^, to Ni^2+^ from 450 to 800 °C and is determined by the nature of the octahedral cation.
Xue and Pinnavaia, 2008 [38]	Modified from Vogels et al., 2005; stoichiometric mixture containing Si/Al^3+^ gel from water glass solution, Al(NO_3_)_3_∙9H_2_O, Mg(NO_3_)_2_∙6H_2_O, urea and water in a molar ratio of 6.6:0.40:3.0:10 per 400 moles of water	Sol-gel co-precipitation at 90 °C for 24 h	saponite, poor crystallinity, high surface area, BJH pore volume and pore size	Has decent transparency when incorporated in glassy epoxy polymer.
Schumann et al., 2012 [39]	56.25% SiO_2_, 4.30% Al_2_O_3_, 30.88% MgO, and 3.97% K_2_O; saponite formula: K_0.33_Mg_3_(OH)_2_(Si_3.67_Al_0.33_)O_10_. The gel is added with NaOH and Na oxalate solutions	Sol-gel precipitation at 60 °C and ambient pressure for 3 months!	predominantly saponite with Mg^2+^ in interlayers and talc byproduct	Gives replicating clay minerals; possibly through template-catalyzed polymerization, charge distribution transmitted from layer to layer.
Besselink et al., 2020 [40]	Modified from Vogels et al., 2005	2-step sol-gel co-precipitation at 25–95 °C at varying synthesis time from 5 min to 90 days	Mg-saponite - nanocrystals;	Two-step saponite crystallization—(1) amorphous aluminosilicate network formation, (2) crystallization of this amorphous aluminosilicate network towards saponite in the presence of magnesium and urea.
Vicente, I. et al., 2010 [41]	slurry (≈9wt.% solids), with a Si^4+^:Al^3+^:Mg^2+^:NH_4_^+^ composition ratio of 14.3:2.5:12.5:8 which would result in saponite with the theoretical formula (NH_4_)_1.2_[Mg_6_Al_1.2_Si_6.8_O_20_(OH)_4_]	Microwave, 180 °C for 6 hrs	NH_4_-saponite; some have higher crystallinity, higher CEC and higher Al(Td)/Al(Oh) ratio of saponites formed	Physico-chemical properties of resulting saponite is influenced by initial slurry pH
Trujillano et al., 2011 [42]	sodium silicate solution (27% wt, d = 1.39g/mL); NaOH and NaHCO_3_; chlorides M^3+^ = Al^3+^ or Fe^3+^, and M^2+^ = Mg^2+^, Ni^2+^, or Fe^2+^; saponite theoretical formula [Si_7_M^3+^][M^2+^_6_O_20_(OH)_4_Na.nH_2_O	Microwave, 180 °C for 8h	saponites containing divalent Mg, Ni or Fe - large surface area	A potentially good oxidation catalyst especially for NiMgAl; possible synergistic effect takes place when two different cations are present in adjacent octahedral positions
Gebretsadik et al., 2015 [43]	modified from Trujillano, et al., 2011 and Gebretsadik et al., 2014	Microwave, 180 °C for 6h	Na-saponite, NH_4_-saponite	Characterized by higher delamination with smaller lamella and higher BET area; higherincorporation of Al in the tetrahedral sheet (higher Al(T)/Al(O) ratio)

Noteworthy is the mild non-hydrothermal synthesis procedure for saponite described by Vogels et al., as it allowed scaled up preparation as well as easy tunability of the texture and composition of the saponites [27,44,45]. The method involves a precursor mixture with a saponite theoretical composition of N_x/z_^z+^[M_6_][Si_8-x_Al_x_]O_20_(OH)_4_⋅nH_2_O. M and N correspond to the divalent octahedral and the interlayer cations, respectively. The octahedral cation, M, is used as prefix to name the saponites, such as Zn-saponite when the octahedral cation is Zn^2+^. The experiments were performed at a constant mild temperature of 90 °C for 20 h (unless otherwise indicated) in a double-walled Pyrex vessel which had stirrer and baffles that ensured homogeneity of the mixture within. A gel with 5.67 (x = 1.2) Si/Al molar ratio was made by diluting Na_2_SiO_3_ solution (27 wt% SiO_2_) in demineralized water and gradually adding under constant stirring a previously prepared Al(OH)_4_^−^ (from Al(NO_3_)_3_⋅9H_2_O in NaOH solution) to form the gel. Several more gels with Si/Al molar ratios of 7.89 (x = 0.9) and 12.3 (x = 0.6) were prepared similarly by adjusting the amounts of the aluminum and silicate sources. In all the experiments, the sum of Si + Al was kept constant. Gels with an exceptionally low Si/Al ratio of 2.33 (x = 2.4), the aluminum nitrate was not added with NaOH to decrease the rate of gelation. However, for a gel with a Si/Al ratio of 39.0 (x = 0.2), the preparation was modified because the authors observed that after mixing the Si- and Al-containing solutions, no gels were formed. Only after addition of an appropriate amount of HNO_3_ (65%) was a stable white gel formed. The gels that were formed were then added to demineralized water, placed in the aforementioned vessel and the temperature increased to 90 °C. Synthesis was started when the gel-water mixture (maintained at 90 °C) was added with the needed amounts of nitrates of M^2+^ cations (M^2+^ = Mg^2+^, Ni^2+^, Zn^2+^, Co^2+^, and Cu^2+^) and urea dissolved in water. When the saponites were desired to have octahedral sheets with two metal cations whose ratio varied from 1 and 29, appropriate amounts of the divalent metal nitrate salts were combined in the synthesis mixtures. The prepared mixtures were not acidified before the start of the synthesis because the gels were unstable in acid conditions. The effect of urea concentration, which was used as the hydrolyzing agent, on saponite formation was investigated by using Zn-saponite as a test sample. In addition, a synthesis was performed where the pH of the initial mixture was 8 (achieved by using urea together with additional NaOH). After synthesis, the suspended solids were filtered, washed thoroughly with demineralized water and dried at 130 °C before characterization. Some of the prepared saponites were exchanged with Na^+^, NH_4_^+^ or Al^3+^ by suspending and stirring in 1 M NaCl, NH_4_Cl, or AlCl_3_ overnight [27].

Similarly, gels with 5.67 (x = 1.2) or 7.89 (x = 0.9) Si/Ga molar ratio and with Mg^2+^ or Zn^2+^ as the octahedral cation were prepared [45]. Ga(OH)_4_^−^ (from GaCl_3_ in NaOH solution) was added gradually while stirring continuously to a diluted Na_2_SiO_3_ solution (27 wt.% SiO_2_) to form the gel. B-containing saponites were also synthesized using the gel method and an “aerosil” method. The latter method involved mixing aerosil from Degussa with borax (Na_2_B_4_O_7_) at a corresponding Si/B molar ratio of 2.9–12.3 in demineralized water. As in previous synthesis, the mixtures were heated to 90 °C, then added with the required amount of Mg(NO_3_)_2_⋅6H_2_O or Zn(NO_3_)_2_⋅4H_2_O, together with Na(NO_3_) and urea. The influence of pH on saponite formation was studied by performing the synthesis at a starting pH level of about 8 and of about 3 (achieved by adjusting with concentrated HNO_3_). The temperature was kept at 90 °C for the whole synthesis duration (20 h). The products were then filtered, washed thoroughly with demineralized water and dried overnight at 130 °C before further analyses [45].

The method by Vogels at al. [27]—which separates the synthesis into two steps: (1) formation of the precursor aluminosilicate gel and (2) hydrolysis of the divalent metal by the slow release of ammonia from the decomposition of synthesis urea—is a reasonable analog of natural processes. The aluminosilicate gels in the first step forms a four-fold-coordinated Al^+3^ similar to what is found in nature such as in volcanic glass. The use of urea, a compound figuring in many prebiotic model reactions, circumvents the formation of brucite, Mg(OH)_2_, in the final saponite product by slowly releasing ammonia thereby controlling the hydrolysis of magnesium [46]. This enables the formation of saponite without the accompanying sudden increase in pH which would have precipitated brucite [40]. 

Research studies making use of the thermal decomposition of urea as a means to control the pH during the synthesis of saponites include those of Besselink et al. [40], Prihod’ko et al. [47], Sychev and Prihod’ko [37], Xue and Pinnavaia [38], and Yu et al. [48]. Given that urea has more potential than just controlling the pH in clay mineral synthesis as it is a potential precursor of nucleobases and related molecules and a promoter of phosphorylation [25,26,49], the urea-assisted synthesis of saponites might be useful as starting point in extending research into urea assisting the synthesis of clay minerals while at the same time providing a source of precursors for nucleobases formation.

### 2.3. Characterization of Synthetic Saponites

Routine characterization techniques for saponites include X-ray Diffraction (XRD), X-ray fluorescence (XRF), Fourier Transform Infrared Spectroscopy (FTIR), magic-angle spinning-nuclear magnetic resonance (MAS-NMR), extended X-ray absorption fine structure spectroscopy (EXAFS) and pore and surface area analysis, thermogravimetric analysis and transmission electron microscopy (TEM). This section discusses how the group of Vogels et al. [27,44,45,50] used these techniques to determine the structure of synthetic saponites and the changes they undergo as they interact with their environment. 

#### 2.3.1. Powder X-ray Diffraction (XRD) 

Although quantitative analysis of clay minerals using XRD remains challenging, due to the various chemical compositions, preferred orientation, structural disorder and great structural diversity of clay minerals, it remains one of the most important analytical approaches used in the qualitative study of clay samples [51]. Typically, diffraction patterns of randomly oriented powder samples of the synthesis products are obtained with a diffractometer using CuKα or CoKα radiation, with alumina as a standard. 

**Figure 2:**
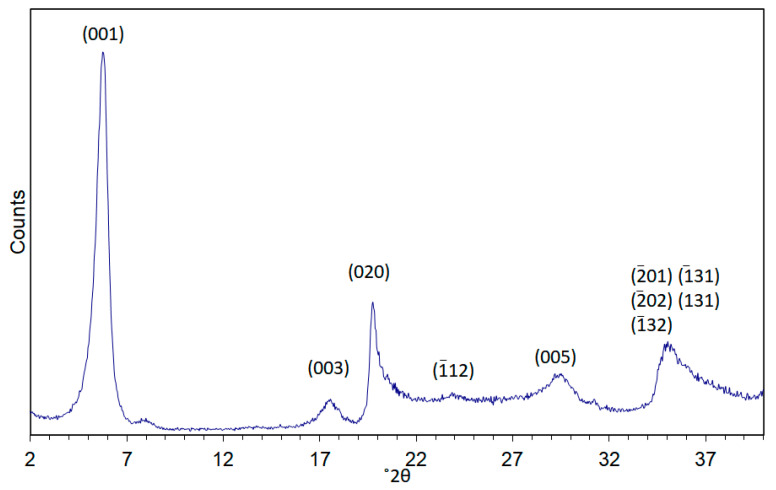
Typical X-ray diffraction pattern of a synthetic Zn-saponite synthesized for 20 h with 72.1 g urea. Based on data from [27]. Note: no vertical axis was given in the original publication.

XRD was used to follow the crystallization and aging of Mg- and Zn-saponites [27]. The XRD pattern of Zn-saponite is shown in Figure 2, for the complete set of XRD patterns, the reader is directed to [27]. The intensities of the (001) reflections of the synthetic saponites increased strongly and sharpened during aging. This points to a higher degree of stacking of the saponite layers. The d-spacing of the (001) reflections decreases slightly from 13.2 to 12.7 Å. This was attributed to either an increase in particle size as larger particle size results in an increasing resistance to expansion, or a consequence of the interaction between the Lorentz-polarization (Lp) factor and the inference function. An intensity increase of the Lp factor with decreasing angle would result in a shift to lower angles of the maximum of broad peaks. From the XRD measurements, it was inferred that within 3 h, the reaction of Mg-saponite proceeded to a substantial extent resulting in platelets (length: 5–10 nm, as observed by TEM) without stacking. Longer synthesis times such as at 20 and 47 h, the Mg-saponite particles formed still consisted of small platelets (15–25 nm) with a low extent of stacking (∼1–4 layers). This was consistent with observed preferential growth in the *a-b* direction of the Mg-smectites prepared at low temperatures [52]. Saponites having a single type of metal ion within the octahedral sheets show XRD patterns with similar positions of the reflections except for differences in the position of the (001) reflections: 15.7 Å for Mg, 14.7 Å for Ni, 14.0 Å for Co, and 12.7 Å for Zn. The different d-value of the (001) reflection is probably a result of particle size effects, although the effect of the presence of the divalent cations within the interlayer could not be ruled out. XRD also revealed that synthesis of Cu-saponite was not successful; chrysocolla [Cu_2_Si_2_O_5_(OH)_2_] was formed instead [27,52]. Variation in the Si/Al ratio from 5.67 to 39.0 was reported to have little effect on the XRD patterns. The very sharp XRD (001) reflection recorded for Zn-saponite suggests the presence of relatively large and crystalline saponite crystals. In contrast, Mg-, Co-, and Ni- saponite were formed in smaller sizes than those of Zn-saponites as revealed by the very broad (06*l*) reflections. This was corroborated by the reported TEM results [27]. 

XRD was also used to follow the swelling behavior of the synthetic saponites by ethylene glycol (EG) vapor equilibration at 50 °C for 4 to 6 days prior to XRD measurements. Only the Mg- and Zn-saponites were explored in [27,45]. Results reveal the ability of the saponites to swell. Na^+^-exchanged Zn-saponite containing Al^3+^ in the tetrahedral sheet exhibited a d(001) value of 12.7 Å, roughly similar to those containing Ga^3+^. Treating the saponites with EG increased the d(001) spacing to 14.8 Å. Ga^3+^ containing Mg-saponite (Na^+^-exchanged) exhibited d(001) spacing of 16.0 Å which increased to 16.5 Å after EG treatment. Determination of the interlayer space in swelling clays is important as it would provide information on what type and size of guest molecules that can be accommodated.

#### 2.3.2. Infrared Spectroscopy (IR)

**Figure 3:**
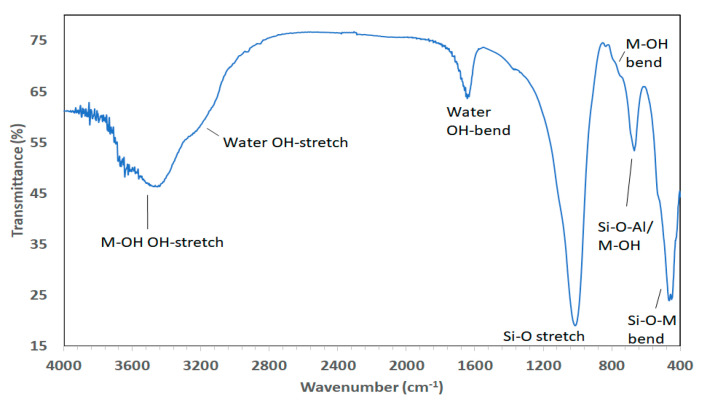
Typical infrared (IR) spectrum of a synthetic Mg-saponite synthesized in 20 h with 72.1 g urea. The IR spectrum was collected using a Perkin Elmer (1600 series) spectrometer in transmission mode using 256 scans at 4 cm^−1^ resolution using a KBr tablet (5 mass% sample).

Infrared (IR) spectroscopic techniques have long been established for the study of the clay mineral frameworks and of molecules adsorbed on their surfaces [53]. In addition, it has been applied to study the formation of clay minerals during synthesis [54]. In the studies by Vogels et al. [27,31] IR spectroscopy was typically performed using an IR spectrometer equipped with an in situ diffuse reflectance infrared Fourier transform (DRIFT) accessory. The samples were normally diluted with dry KBr (approximate 5 mass% sample) and ground in a mortar and the measurements performed at room temperature. All spectra were generally recorded by accumulating 256 scans with a resolution of 4 cm^−1^, unless longer scans were necessary. The obtained spectra were ratioed against background vibrations and corrected for KBr absorption. The spectra of natural saponites (e.g., from Krugersdorp, Transvaal) (compiled by H.W. Van der Marel and H. Beutelspacher in Atlas of Infrared Spectroscopy of Clay Minerals and Their Admixtures [55] and cited by Kloprogge et al. [56]) revealed bands at 3675 (Mg−O−H), 3625 (Al−O−H), 3420 (H−O−H), 1630 (H−O−H), 1103 (Si−O), 1058 (Si−O/(Si−O−Si), 1005 (Si−O−Al), 750 (Si−O−Al), 693 (S-O−Mg), 652 (Mg−OH), 610 (Si−O), 528 (Si−O−Al/Mg), 480 (Si−O), 462 (Si−O−Mg), and 449 cm^−1^ (Si−O−Mg). The spectra of the synthetic saponites (Figure 3) described by Kloprogge et al. [28,56] agreed well with the values observed for the natural saponite from Krugersdorp, although some Si−O vibrations were very weak or even absent. Upon exchanging the interlayer cation for NH_4_^+^ a distinct additional band became visible at 1430 cm^−1^, in agreement with the findings of Kloprogge et al. [13] for synthetic ammonium-saponite. All other types of interlayer cations as well as the Si/Al ratio (5.67 to 39.0) did not affect the IR spectra. Ni-saponites exhibited one additional sharp absorption band at 2183 cm^−1^ which was assigned to an antisymmetric stretching vibration of N=C=O groups. Apparently, some isocyanate, which is an intermediate product during the hydrolysis of urea, had bonded to Ni^2+^-OH through a hydrogen bond, possibly by adsorption at the edge of the saponite octahedral sheet. After ion exchange with NH_4_^+^, Ni-saponite hardly exhibited an absorption band corresponding to (interlayer) NH_4_^+^, indicating a very low layer charge. The absorption bands of other NH_4_^+^ exchanged saponites with similar Si/Al ratios all showed an intense and sharp band around 1430 cm^−1^ indicating higher layer charges. The very low layer charge of Ni-saponite was explained by substitution of Ni^2+^ by Al^3+^ in the octahedral sheet. The charge deficiency caused by the isomorphous substitution in the tetrahedral sheet was probably almost completely compensated by the octahedral substitution, because a high fraction of the Al^3+^ was incorporated in the octahedral sheet. Zn-saponites exhibit an opposite behavior compared to Ni-saponites. The intensity of the NH_4_^+^ absorption band increased with the amount of Al^3+^, even when an “excess” amount of Al^3+^ was used (Si/Al = 2.33). Even though a higher amount of Al^3+^ resulted in an increasing percentage of six-fold coordinated Al^3+^ in the octahedral sheet (up to 40% for Si/Al = 2.33), the layer charge seemed to increase, which favored the (muscovite) substitution 3Mg^2+^ ↔ 2Al^3+^ + vacancy, creating no positive charge on the octahedral sheet to compensate for the negative charge on the tetrahedral sheet [28].

#### 2.3.3. Transmission Electron Microscopy (TEM)

Electron microscopy (EM) including transmission electron microscopy (TEM) is one of the irreplaceable techniques for the investigation of clay minerals providing useful pieces of structural, physical, and chemical information (when equipped with energy or wavelength dispersive X-ray detector), which are not obtainable by other techniques. Typically, powdered samples are dispersed in ethanol (96 vol%) and ultrasonically treated for 5 min. A drop of the resulting suspension is then placed on a holey carbon film supported by a copper grid and the solvent evaporated. The samples were then investigated using a transmission electron microscope operated at an accelerating voltage of 100–200 kV. Coupled with energy-dispersive X-ray (EDX) analyzers, chemical analysis of samples can be performed. For example, an Al^3+^ exchanged MgZn-saponite was analyzed to obtain information about the incorporation of both Zn^2+^ and Mg^2+^ in the clay structure [27,57]. The samples after 50 min of synthesis showed clusters of exceedingly small spherical particles typical of the starting Zn-Si/Al gel. TEM revealed that Zn-saponite formed platelets (length 15 nm) with little stacking in between the remaining gel particles after 1.5 h of synthesis. After 12.25 h, the synthesis was completed and clay platelets with a length of 100 to 200 nm consisting of stacks of about 10 layers were formed. Increasing synthesis hours to 22.75 h resulted in particle ripening as indicated by a decrease in the number of particles but increase in particle size [27,57].

#### 2.3.4. Magic-Angle Spinning-Nuclear Magnetic Resonance (MAS-NMR) Spectroscopy

High resolution magic-angle spinning-nuclear magnetic resonance (MAS-NMR) spectroscopy of solids is a powerful tool for understanding the fine structure of clay minerals. For example, the precursor Si/Al gel and the saponite products of the urea-assisted synthesis by Vogels et al. [27,45] were characterized by ^27^Al and ^29^Si MAS-NMR spectroscopy. This allowed for the observation of the transformation of the Si and Al local environments. ^27^Al MAS-NMR spectra were collected using the following parameters: 130.321 MHz with a pulse length of 1 µs and a pulse interval of 1 s while ^29^Si MAS-NMR spectra were collected at 99.364 MHz with a pulse length of 6.5 µs and a pulse interval of 40s. Chemical shifts (δ) of ^27^Al and ^29^Si were reported in ppm relative to [Al(H_2_O)_6_]^3+^ and [(CH_3_)_4_Si], respectively.

In addition to saponite with different divalent metals on the octahedral position, saponites were synthesized in which tetrahedral metals were substituted by Ga and B [45]. Chemically, gallium reacts similarly to aluminum while boron can substitute for silicon. The XRD pattern of the Ga- and B-substituted saponites were similar to Al-substituted saponites. ^11^B MAS-NMR experiments were executed at 160.466 MHz with a pulse length of 0.8 μs and a pulse interval of 0.25 s. ^71^Ga MAS-NMR experiments were performed at 152.531 MHz with a pulse length of 2.0 μs and a pulse interval of 0.5 s. Chemical shifts (δ) of ^11^B and ^71^Ga were reported in ppm relative to [BF_3_(OEt_2_)] and [Ga(H_2_O)_6_]^3+^, respectively [45]. 

**Figure 4:**
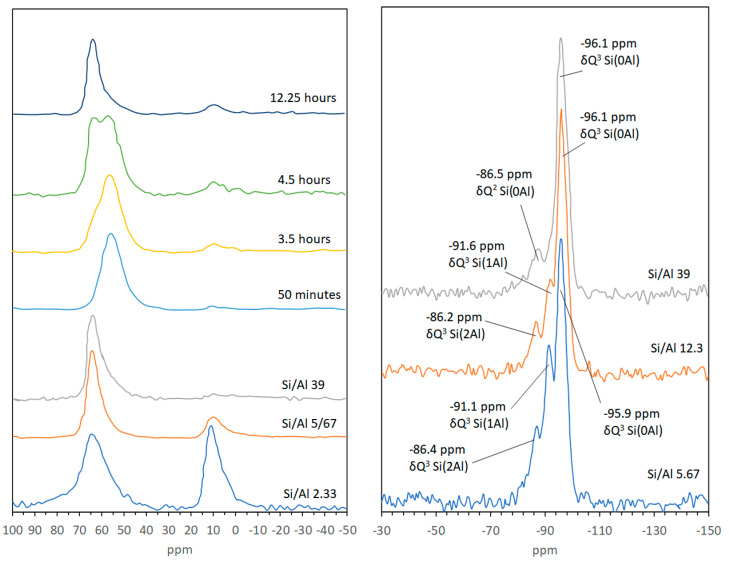
Magic-angle spinning-nuclear magnetic resonance (MAS-NMR) of Zn-saponite with varying Si/Al ratio and varying synthesis time. ^27^Al MAS-NMR (left) were performed at 130.321 MHz with a pulse length of 1 µs and a pulse interval of 1 s and chemical shifts (δ) reported in ppm relative to [Al(H_2_O)_6_]^3+^. ^29^Si MAS-NMR (right) was performed at 99.364 MHz with a pulse length of 6.5 µs and a pulse interval of 40s and chemical shifts (δ) reported in ppm relative to [(CH_3_)_4_Si]. Modified from [27].

^27^Al MAS-NMR confirmed that Al^3+^ in the Si/Al gel is situated in fourfold coordination, as reflected by a single resonance at approximately 56 ppm. ^27^Al MAS-NMR was also used to follow the crystallization and aging of Zn-saponite (Figure 4) [27]. A broad band at 56 ppm corresponding to the Al^4^ of the Si/Al gel was observed in the first 1.5 h of synthesis. After 3.5 h, some new resonances appeared next to the main gel resonance at 56 ppm. These resonances can be attributed to Al^6^ visible at about 9 ppm together with an Al^4^ shoulder detectable at approximately 63 ppm indicative of Al surrounded by three nearest-neighbor Si atoms. The signals most likely originated from the saponite structure. A further increase of the ageing time clearly resulted in the strong rise of the Al^4^ saponite resonance at 63 ppm and a corresponding decrease of the Al^4^ peak of the Si/Al gel at 56 ppm. In addition, the Al^6^ signal increased slightly during the first hours of the synthesis. ^27^Al and ^29^Si MAS-NMR experiments can also be performed to study in detail the effect of the Si/Al ratio on the incorporation of Al^3+^ in the saponite structure. Lowering the amount of Al^3+^ clearly resulted in a decrease of the Q^3^ Si(1Al) resonance (i.e., a Si connected to 2 Si and 1 Al next nearest neighbors), and to a smaller extent of the Q^3^ Si(2Al) resonance (Si connected to 1 Si and 2 Al), as shown by the ^29^Si MAS-NMR spectra of Zn-saponites prepared with Si/Al ratios of 12.3 and 39.0. The relatively high intensity of the resonance around about −86 ppm in saponites with a Si/Al ratio of 39.0 is unlikely to be due to Q^3^ Si(2Al) taking into account the low amount of Al^3+^ and the nearly completely disappeared Q^3^ Si(1Al) resonance. A better explanation for the peak at −86 ppm is Q^2^ Si(0Al) present at the clay edges. The synthetic saponites are composed of exceedingly small particles with consequently a high amount of Si^4+^ situated at the clay edges. The fact that this Q^2^ Si resonance in ^29^Si MAS-NMR spectra of clay minerals is usually not observed in the literature can possibly be explained by the relatively large particle size of these (natural) samples compared to the synthetic saponite samples [27]. 

**Figure 5:**
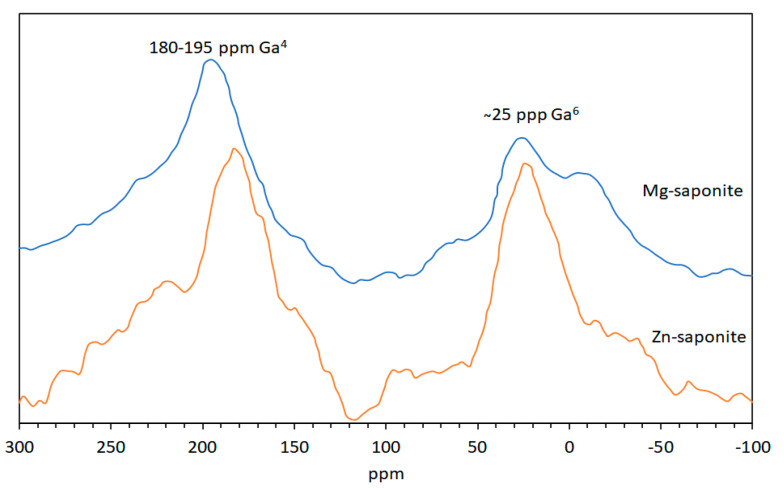
^71^Ga MAS-NMR of gallium substituted Mg- and Zn-saponite performed at 152.531 MHz with a pulse length of 2.0 μs and a pulse interval of 0.5 s and chemical shifts (δ) reported in ppm relative to [Ga(H_2_O)_6_]^3+^. Modified from [45].

The ^71^Ga MAS-NMR spectra (Figure 5) of Ga^3+^ containing Mg- and Zn-saponite exhibit two broad peaks at ~25 and 180–195 ppm. As it is expected that, like Al^3+^, Ga^3+^ can be found in both the tetrahedral (Ge^4^) and octahedral (Ge^6^) coordination sites of saponite, it would be reasonable to attribute the two broad peaks to Ga^3+^ in these positions with the lower chemical shift assigned to Ga^6^ [58]. The position of the Ga^4^ resonance of Mg-saponite is about 10 ppm more positive than that of the Ga^4^ resonance in Zn-saponites. This is due to the higher ditrigonal rotation angle α in the tetrahedral sheets as a result of the smaller octahedral sheets in Mg-saponites. This is consistent with the observed shift to higher values of the peaks assigned to tetrahedral cations for related Al^3+^ containing 2:1 phyllosilicates when the rotation angle α increased [59]. 

**Figure 6:**
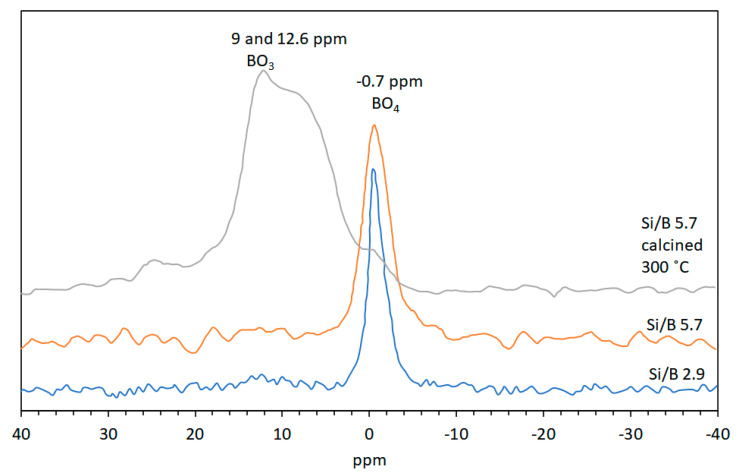
^11^B MAS-NMR of Mg-saponite with different Si/Al ratios and after calcination at 300 °C. ^11^B MAS-NMR were run at 160.466 MHz with a pulse length of 0.8 μs and a pulse interval of 0.25 s and chemical shifts in ppm relative to [BF_3_(OEt)_2_]. Modified from [41].

^11^B MAS-NMR typically shows the resonance from trigonal B^3+^ (BO_3_) at around 19ppm and tetrahedrally coordinated B^3+^ (BO_4_) at about 2 ppm [60]. Quadrupole coupling differences results in BO_3_ units usually appearing as broad doublets, whereas BO_4_ peaks are sharp [61]. A shift to a higher field of the BO_4_ peak has been shown to be a result of boron cations within silicate lattices. For example, the BO_4_ peak for danburite (CaB_2_Si_2_O_8_), B^3+^ containing alkali feldspar (NaAlSi_3_O_8_) and B^3+^ in zeolites is at −0.7 ppm, −1.1 and −2.5 ppm, and −3 to −5 ppm, respectively [60,62]. Vogels et al. [45] collected ^11^B MAS-NMR spectra of the Si/B gels and showed that it only displayed a sharp signal at −1.7 ppm consistent with BO_4_ coordinated with SiO_4_ tetrahedra. This NMR pattern of the Si/B gel was not sensitive to hydration and drying effects as the signal at −1.7 ppm for gels at 25 °C shifted only slightly to −1.9 ppm when the gels were calcined at 300 °C. Boron containing Mg-saponite synthesized from the gel with Si/B molar ratio of 1.1 exhibited a broad peak of low intensity between 12.6 and 9.0 ppm and a sharp peak at −0.8 ppm. Mg-saponite with Si/B ratio of 2.9 and 5.7 exhibited only a sharp ^11^B MAS-NMR signal at −0.4 and −0.7, respectively (see Figure 6). Albeit small, the shift of the BO_4_ signal of the synthetic saponite was significant as compared to the gel. This was taken as an indication of a successful incorporation of B^3+^ in the tetrahedral sheet of the saponite lattice. Calcination at 300 °C clearly showed the disappearance of the −0.7 ppm signal and appearance of the broad peak between 9 and 12.6 ppm. This indicated the transformation from BO_4_ to BO_3_. Rehydrating the calcined saponite overnight at room temperature showed that the B^3+^ reverted back to tetrahedral coordination. This change in coordination of B^3+^ during dehydration/rehydration processes had been observed for B-containing boralite [63].

#### 2.3.5. Nitrogen Physisorption Measurements

The role of clay minerals in the origin of life hinges on their ability to adsorb prebiotic molecules and subsequently catalyze their reactions into complex molecules. To understand the underlying mechanisms of these processes, the surface properties of clay minerals have to be precisely determined. Among the surface properties, the specific surface area (SSA) is a crucial parameter as it provides quantitative assessment of the areas available for surface reactions. The SSA of non-swelling and non-microporous clay minerals range from a fraction to more than 100 m^2^/g. Higher values are obtained with microporous clay minerals and swelling (expanding) clay minerals [64].

Vogels et al. [27] obtained specific surface areas and (micro-) pore volumes (according to IUPAC conventions [65]) from nitrogen adsorption-desorption isotherms at −196 °C using a surface area and porosimetry instrument. Adsorbent outgassing protocols were followed by exposing the powdered samples at temperatures around 130 °C under vacuum [27,65]. The total surface areas are calculated using the Brunauer-Emmett-Teller (BET) equation [66], and micropore surface areas are determined from t-plots, the external surface areas being the difference between the BET and micropore surface areas [67]. 

**Figure 7:**
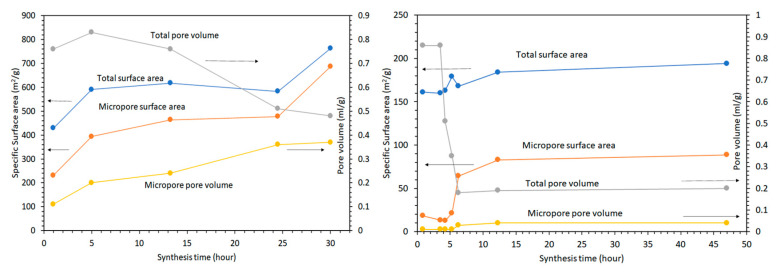
BET total and micropore surface area and pore volume of Mg- (**left**) and Zn-saponite (**right**) as function of synthesis time. These results are calculated based on Figure 5 in [27].

N_2_ adsorption-desorption isotherms for Mg-saponites synthesized for 5 to 30 h by Vogels et al. [27] were constructed and the BET total and micropore surface area and pore volume results are shown in Figure 7 (left). Synthesis for just 5 h exhibited an H1 type of hysteresis loop reflecting the gel matrix, similar to the gel matrix of Zn-saponite (vide infra) after 50 min preparation [27,65]. An H1 type of hysteresis is characterized by adsorption-desorption branches that are almost vertical and almost parallel over a wide range of gas uptake and is exhibited by materials with regular even pores with no interconnecting channels [65], such as MCM-41 (Mobil Composition of Matter No. 41) and SBA-15 (Santa Barbara Amorphous 15) [68]. Increasing the synthesis time to 24.5 h produced Mg-saponite exhibiting a type H2(b) hysteresis loop in accordance with the 2015 updated IUPAC classification [65]. The H2 pattern in the 1985 IUPAC classification [69] is now labeled H2(a) [65]. It is characterized by a sloping adsorption branch and nearly vertical desorption branch. The steep desorption of the H2(a) loop is associated with pore-blocking or percolation in a narrow range of pore necks. It can also be associated with cavitation-induced evaporation. Type H2(b) hysteresis loop is also associated with pore-blocking but the percolation is through a wider neck width distribution. This hysteresis pattern has also been observed in hydrothermally treated SBA-16 [70]. Further increasing synthesis time to 30 h produced Mg-saponite powder that exhibited H4 type hysteresis loops which features an adsorption branch that resembles a composite of Types I and II isotherms and stays nearly horizontal over a wide p/p° range [65]. The H4 pattern is normally interpreted as due to filling of micropores and exhibited by micro- and mesoporous materials such as some zeolite foam series of Pt-catalyst supports [71] and in micro-mesoporous activated carbon [72]. The considerably higher amount of micropores noted for Mg-saponites in relation to Zn-saponites synthesized by Vogels et al. [27] is suggested to be related to pH effects. This was argued based on the fact that after addition of the solution of bivalent metal ions some precipitation (of hydroxides) proceeded with Ni, Co, and Zn and a consequent decrease in pH. Since the interaction and also the size of the elementary particles of a M^2+^/silica/alumina suspension are strongly influenced by the pH, the clustering, and the size of the particles during the initial period of the synthesis will be, with the preparation of Mg-saponite, different from that found with the synthesis of the other saponites [27]. For comparison, isotherms of Zn-saponites with synthesis times of 0 to 3.5 h were also constructed and the BET total and micropore surface area and pore volume results are shown in Figure 7 (right). As discussed above, the hysteresis loop of these specimens is of the H1 type of the IUPAC classification. This pattern is typically found for agglomerations of porous materials with a narrow size distribution of cylinder-shaped mesopores. As with Mg-saponite (vide supra), longer synthesis times showed a distinct change from the H1 isotherm (3.5 h) to the mainly H4 isotherms found for the saponites aged for at least 6.25 h. This corroborated with their TEM observations of the Si/Al gel, which showed formation of clusters of very small spherical particles (after 50 min of synthesis) to formation of clay platelets which were 100–200 nm in lengths and stacked to about 10 layers [27]. The total surface area of Zn-saponites is 194 m^2^/g for samples synthesized for 47 h, in contrast to the 763 m^2^/g total surface area of Mg-saponites synthesized for 30.0 h [27]. A related saponite clay synthesized by Xue and Pinnavia in 2008 based on the methods of Vogels et al. [27] but using water glass as source of silica formed aggregates of irregularly stacked tactoids with a surface area of 920 m^2^/g [38]. A high surface area of a clay mineral can provide more adsorption sites and thus allow concentration of monomers and their subsequent polymerization. It is in this context that montmorillonite, an abundant swelling clay mineral, had been used as a model system for the adsorption and polymerization studies of organic molecules related to the origin of life [4,6,7,8,13,21]. However, most of these studies report a number of adsorbed nucleotides (in mol) per gram of modified swelling clays and cannot be correlated with the total surface area of a clay mineral. It would be beneficial if quantities adsorbed are normalized to the specific surface areas of adsorbents, as proposed by Pedreira-Segade et al. [73]. 

#### 2.3.6. X-Ray Fluorescence Spectroscopy (XRF) 

X-ray fluorescence (XRF) is normally used for routine, relatively non-destructive chemical analyses of rocks, minerals, sediments, and fluids. The instrument works on wavelength-dispersive spectroscopic principles but is typically applied for analysis of bulk specimens. XRF involves easy and inexpensive sample preparation and when the stability and ease of use of X-ray spectrometers is added into consideration, XRF becomes one of the most widely used methods for analysis of major and trace elements in rocks, minerals, and sediment [74].

**Figure 8:**
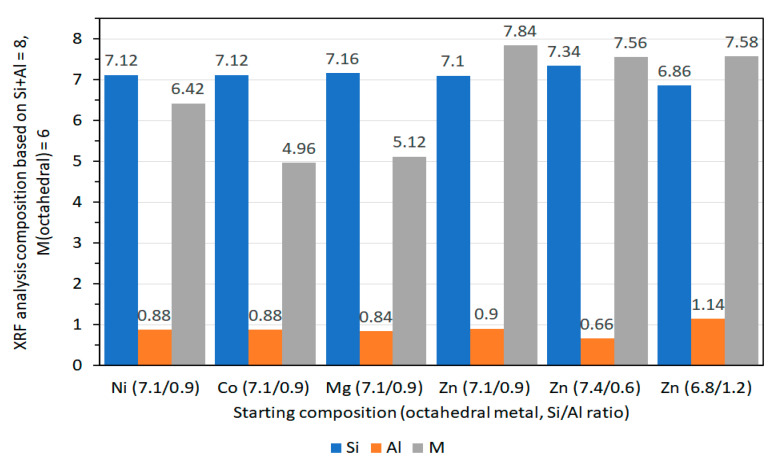
X-ray fluorescence (XRF) analyses of different M-saponites (M = Ni, Co, Mg, Zn) and Zn-saponite with different Si/Al ratios. Results based on Table 5 of [24].

XRF measurements of saponite powdered samples were collected using an automated X-ray fluorescence spectrometer to obtain information about the bulk chemical composition of the samples. The XRF data of Vogels et al. [27], presented in molar ratios reflects good agreement of the Si/Al ratio of the resulting synthetic saponites with the intended Si/Al ratio for all types of octahedral cations (Figure 8). The experimentally determined fraction of octahedral cations slightly deviates from the theoretical value of M = 6 in $N_{x/z}^{z+}$ [M_6_][Si_8-x_Al_x_]O_20_(OH)_4_·nH_2_O. The lower octahedral cation concentrations for Mg- and Co-saponite (5.12 and 4.96, respectively) compared to the theoretical value of 6 is suggested to be due to incomplete crystallization with some gels remaining as observed with TEM for Mg-saponites. The incomplete crystallization for Mg^2+^ was rationalized in terms of solubility of Mg^2+^ at the final pH (around 7) after 20 h of synthesis. Meanwhile, the low value for Co^2+^ concentration in Co-saponite had been argued as due to complexation of Co^2+^ with ammonia and some precipitation as Co(OH)_2_, the latter being observed with temperature-programmed reduction (TPR) experiments. As for the complexation of Co^2+^ with ammonia, this can occur in aqueous ammonia but the resulting [Co(NH_3_)_6_]^2+^ is readily oxidized in this medium. The preferred ligand of Co^2+^ in this case, would be H_2_O while ammonia complexes preferentially with Co^3+^ [75]. The higher value of Zn- and Ni-contents than theoretically calculated in Zn- and Ni-saponites, on the other hand, were attributed to losses of the Si/Al gel during the synthesis process [27]. Notwithstanding the slight deviations in resulting and expected values in chemical composition of the saponites reviewed in this section, XRF remains one of the routine analysis in following synthesis of clays and was used by Prihodko et al. [47], in determining agreement of chemical composition of Mg-, Ni-, and Co-saponite like materials, and by Hongping et al. [76] in determining chemical composition of a series of synthetic saponites, with varying Si/Al ratios.

#### 2.3.7. Extended X-Ray Absorption Fine Structure Spectroscopy (EXAFS)

EXAFS is a popular nondestructive tool for the determination of local atomic structure in a variety of materials. It is the only spectroscopic technique that determines electronic and structural properties of catalysts under reaction conditions and in the presence of reactants [77]. In this section, the use of EXAFS by Vogels et al. [27] to probe the structures of H^+^-exchanged saponites with Mg^2+^, Ni^2+^, Co^2+^, or Zn^2+^ as octahedral cations are highlighted. The saponites are designated as HM-saponite where M is the octahedral metal cation (e.g., HNi-saponite). The EXAFS measurements were performed with a soft XAFS station 3.4 of the SRS facility at Daresbury, UK, which is equipped with a quartz double-crystal monochromator and collimating mirrors that minimize the harmonic contamination of the X-ray beam. EXAFS data at the Al K edge (1559 eV) with a resolution of 1.5 eV were simultaneously collected with a fluorescence detector. The spectra were collected in six scans averaged to minimize both high- and low-frequency noise. The authors collected their EXAFS data in fluorescence mode as the instrumental background in the EXAFS region of electron yield spectra was unreliable. The choice of reference compounds, experimental conditions, and standard procedures for analysis of XAFS data followed the report of Koningsberger and Miller [78]. The latest version of a XAFS Data Analysis Program (XDAP) at the time of the study was used, which allowed for fitting in r-space using both the imaginary and the absolute part of the Fourier transform. This would allow for reliable resolution of the two different Al-O coordination as the imaginary part is much more sensitive to the coordination distance as will be discussed later. Results presented in Vogels et al. [27] showed that for Al^3+^ in saponites, the position of the whiteline for tetrahedral Al is at 2 eV and for octahedral Al is at 6 eV beyond the edge which were sufficiently different that a clear distinction can be made between Al_tet_ and the Al_oct_. The authors have shown that the octahedral contribution decreases in the order Ni > Zn > Mg = Co. However, the intensity of the Al_tet_ whiteline did not increase in the same order. These results were interpreted based on XAFS studies of zeolites which have shown that an increasing whiteline intensity (i.e., increasing positive charge) is related to an increasing acid strength [79]. The EXAFS spectra showed marked differences in the imaginary parts of the Fourier transforms between 1.5 and 2.2 Å and in the higher shells between 2.5 and 3.5 Å. These differences were attributed to different second-nearest neighbors (Ni vs. Mg). From the whiteline data, the authors deduced that both tetrahedrally and octahedrally coordinated Al^3+^ ions were present in the saponite samples. Moreover, information on Al-O coordination distances was reported to be in the range 1.65 to 1.74 Å for Al_tet_ and 1.84 to 1.88 Å for Al_oct_. These are typical for Al-O coordination distances observed for Y zeolites [80] and clay minerals [81]. 

Apart from information on Al-O coordination and bond lengths as well as influence of second nearest neighbors as presented by Vogels et al. [27], EXAFS was used to determine interatomic distances and local environment of other clay minerals. For example, it was used to determine interatomic distances between octahedral cations, Mg, Al and Zn with O in montmorillonite synthesized via fluorine route by Reinholdt et al. [82]. The group also used EXAFS to reveal (1) strong local octahedral sheet distortions with respect to the ideal montmorillonite structure and (2) lengthening of the out-of-plane Zn-Si(Al) distances indicating the swelling of the entire layer in the neighborhood of Zn [82].

#### 2.3.8. pH Analysis 

The pH of the suspensions during saponite synthesis was followed using a pH meter, such as a Schott-Geräde pH-meter CG804 with electrodes from Ingold. Slurry pH measurement cannot be neglected since it can affect the synthesis of saponites [27,29,40,46,83]. For example, Bahldermann et al. [84] observed that high initial slurry pH precipitated brucite, Mg(OH)_2_. This undesired formation of brucite was avoided saponite synthesis was performed in two steps: formation of an aluminosilicate gel at high pH ≈ 13; followed by a saponite formation between pH 7 and 8, aided by hydrolyzing urea [27,46]. The nucleation and growth of the saponite proceeds gradually, and no temporarily fast OH-consumption is exhibited. The initial pH decrease is probably a result of the possible formation of hydroxides. An observed large difference in initial pH-levels recorded with the synthesis of Mg-saponite, on one hand, and the Zn-, Co-, or Ni-saponites, on the other, is due to the different solubilities of the corresponding hydroxides: Mg-hydroxide being significantly more soluble than Ni-, Co-, and Zn-hydroxides (K_sp_ values of the hydroxides are: Mg(OH)_2_ = 5.61 × 10^−12^; Ni(OH)_2_ = 5.48 × 10^−16^; Co(OH)_2_ = 5.92 × 10^−15^; and Zn(OH)_2_ = 3.0 × 10^−17^).

## 3. Thermal Properties of Saponites

### 3.1. Thermal Stability 

The thermal stability of clay minerals is an important parameter to measure since most industrial applications involving heterogeneous catalysis take place at increased temperatures. Components of primitive atmosphere which are soluble in water would react to form various amino acids under hydrothermal conditions. This was shown plausible by Marshall [85], when he synthesized several amino acids and abundant amines from aqueous NH_4_HCO_3_ solutions and C_2_H_2_, H_2_, and O_2_ (formed in situ from CaC_2_, Ca, and H_2_O_2_) at 200–275 °C. Moreover, some studies on the polymerization of glycine with clay minerals were reported under hydrothermal conditions (5–100 MPa, 150 °C) [86]. Therefore, it is important that the catalyst is stable at those temperatures. In the works of Vogels et al. [44], thermal stability was studied using thermal gravimetric analysis (TGA) and differential thermal analysis (DTA). The samples were typically ground thoroughly in air and subsequently calcined in air in a furnace with a constant heating rate of 5 °C/min. Collection of thermograms were performed by placing samples in furnace at room temperature and then raising it to the desired calcination temperature, which ranged from 120 to 900 °C, for 4 or 16 h depending on the subsequent characterization techniques. 

**Figure 9:**
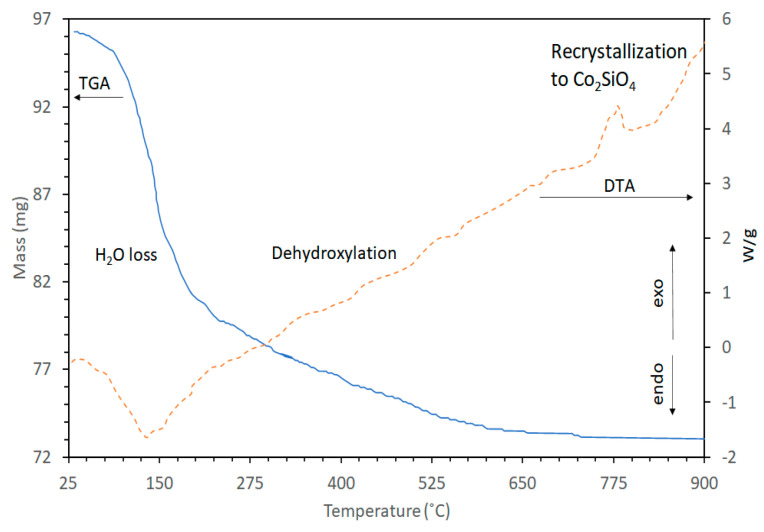
Thermogravimetric (TGA) and differential thermal analysis (DTA) of Co-saponite (Al^3+^ exchanged). Modified from Figure 1 [44].

The curves of mass loss and heat flow versus temperature of Co-saponite studied in [44] is shown in Figure 9. This is comparable to those of the different M^2+^-saponites in the same study. Removal of weakly bound water was exhibited by the sharp mass loss between room temperature and approximately 200 °C in the TGA curve and a corresponding endothermic peak in the DTA curve with a maximum at 130 °C. Removal of the remaining strongly bound interlayer water occurred between 200 and roughly 400 °C and this was followed by the dehydroxylation of the saponite structure beyond approximately 400 °C. No further mass loss was observed at higher temperatures. The dehydroxylation of the synthetic saponites of this study is characterized by a gradual mass loss and lacking an endothermic peak in the DTA curve. This pattern is different from the observed dehydroxylation of other saponites in which the TGA plot showed a second sharp mass loss at a temperature between 500 and 800 °C and an associated clear endothermic peak in the DTA curve [28]. Here, the DTA curve displayed a sharp exothermic maximum at 770 °C (Figure 9) corresponding to the rapid recrystallization of the Co-saponite into Co_2_SiO_4_. 

**Figure 10:**
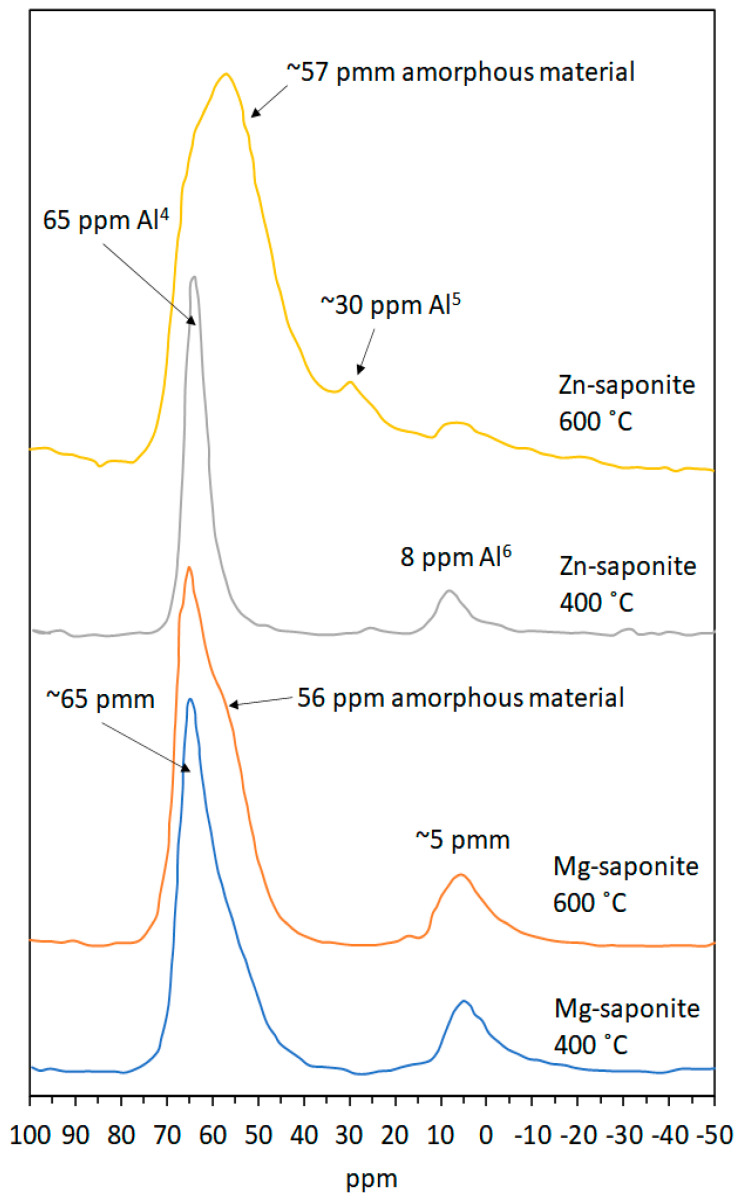
^27^Al MAS-NMR of Mg- and Zn-saponite thermally treated for 4 h at 400 and 600 °C. Redrawn from Figure 2 in [44].

To follow changes in the structure of synthetic saponites upon calcination, Vogels et al. [44] performed thermal analyses alongside MAS-NMR. Figure 10 shows ^27^Al MAS-NMR results obtained with Mg- and Zn-saponite calcined at 400 and 600 °C for 4 h, respectively. Spectra of fresh samples were not shown by the authors as there was no change in the MAS-NMR spectra of a fresh sample compared to a sample calcined at 400 °C for 4 h. The six-fold (Al^6^) and four-fold (Al^4^) coordinated aluminum in the octahedral and tetrahedral sheets of the Mg-saponite lattice are centered respectively at approximately 5 and 65 ppm in the ^27^Al MAS-NMR spectra [44,87]. Increasing the calcination temperature to 600 °C resulted in the appearance of a shoulder at 56 ppm on the low-field side of the Al^4^ saponite resonance, which was interpreted as Al^4^ present in amorphous material. Similarly, the Al^6^ and Al^4^ coordinated aluminum in Zn-saponite calcined at 400 °C are centered at about 8 and 64 ppm in the ^27^Al MAS-NMR spectra. However, increasing the calcination temperature to 600 °C destroyed the structure of Zn-saponite. This was according to the ^27^Al MAS-NMR results where a large Al^4^ resonance at 57 ppm together with a fivefold coordinated aluminum resonance at 30 ppm were suggested to be derived from amorphous materials [88]. Indeed, their XRD results no longer showed crystalline species in the Zn-saponite sample thermally treated at 600 °C. The instability of Zn-saponite compared with Mg-saponite was rationalized based on the strong influence of the composition and the effective ionic radius of the cations in the octahedral sheet on the thermal stability. Hazen and Wones [89] explained that the size of the octahedral cation of trioctahedral micas affects the dimensions in the direction of the *b*-axis of the unit cell and, consequently, the lateral fit (achieved by contracting the tetrahedral sheet, thereby introducing a tetrahedral layer rotation over the angle α) between the tetrahedral and the octahedral sheets. Using the works of Hazen and Wones [89] on trioctahedral micas, which are structurally analogous to saponites, as guide, Vogels et al. [44] reported the ionic radii of the octahedral cations Ni^2+^, Mg^2+^, Co^2+^, and Zn^2+^ present within phyllosilicates to be 0.69, 0.72, 0.74, and 0.75 Å, respectively, and related this to the observed differences in stability of the saponites. The value of α is approximately 9° for a relatively small octahedral cation, such as Ni^2+^. This value will decrease with an increase of the ionic radius eventually reaching 0° when the octahedral cation is sufficiently large. Increasing further the size of the octahedral cation will result in an unstable system, since the tetrahedral sheet cannot adjust further by rotation. The limiting value of the ionic radius is approximately 0.76 Å for α = 0°; this is very close to the size of Zn^2+^ (0.75 Å) in the octahedral sheet, hence, the purported reason for the instability of Zn-saponite. Resistance against thermal decomposition is improved when a large cation is combined with a smaller cation in the octahedral sheet as was observed for MgZn-saponite, which was found to be more thermally stable than pure Zn-saponite [44].

**Figure 11:**
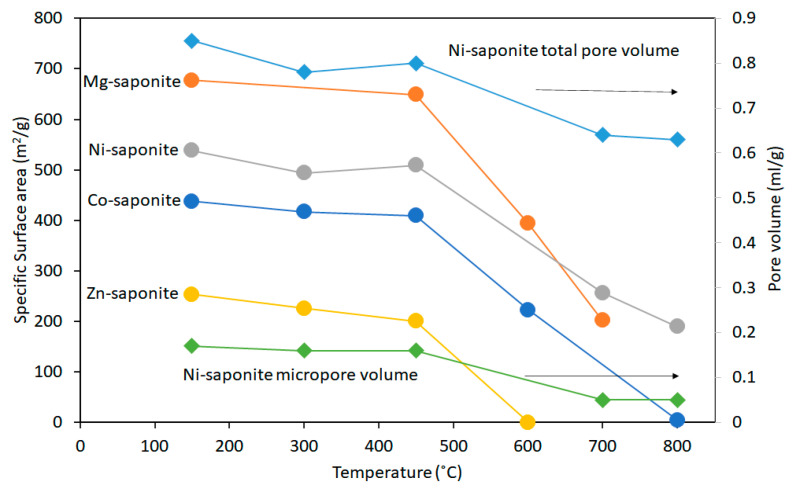
BET surface area and pore volume as function of calcination temperature of M-saponites (M = Mg, Ni, Co, Zn). Modified from Figure 3 in [44].

Vogels et al. [44] also analyzed the development of the specific surface areas (SSA) and pore volumes of some thermally treated saponites (Figure 11). All the saponites calcined at temperatures above 450 °C suffered a partial collapse of the layered structures causing a decrease of the SSA values. Most notable was the decrease of the SSA of Zn-saponite to almost zero at 600 °C. This was attributed to the total breakdown of the layered structure to amorphous material as discussed (vide supra). The total and micro pore volumes of a Ni-saponite thermally treated at different temperatures show hardly any decrease up to 450 °C (Figure 11). As with SSA, the micropore volume decreased at higher temperatures. This was observed for the other saponites, except for Zn-saponite, the total pore volume of which is zero after treatment at 600 °C [44]. Notwithstanding the observed collapse of saponites when treated to temperatures above 450 °C, it is worth noting that the samples remain stable against thermal decomposition at temperatures (200–275 °C) used for hydrothermal synthesis of amino acids from reactants possibly present in primitive environments [85]. 

### 3.2. Hydrothermal Stability

Early sediments can react with hydrothermal water to form clay minerals. Moreover, clay minerals are known to occur at hydrothermal vents and provide important clues to understanding adsorptive synthesis of prebiotic molecules as only in their presence can certain chemical reactions occur [90]. In this featured work of Vogels et al. [44], hydrothermal stability studies are presented for synthetic Mg-saponite and a MgZn-saponite samples. Typically, 100 mg of samples were placed in a quartz reactor, the temperature of which was raised linearly from room temperature to 400, 500, or 600 °C at a rate of 5 °C/min. At the same time, a N_2_ flow of 50 mL/min was passed through the sample. When the desired temperature (400, 500, or 600 °C) was reached, 30 vol% H_2_O in N_2_ (obtained by passing the N_2_ flow through a saturator kept at 70 °C) was put through the sample for 4 or 16 h. The two ^27^Al MAS-NMR resonances at approximately 65 and 5 ppm, which were attributed to aluminum in tetrahedral (Al^4^) and octahedral coordination (Al^6^), respectively, remain visible without displaying a change in chemical shift, even after four hours at 600 °C in steam. This indicated the high stability of the clay structure with respect to hydrothermal treatment. XRD and MAS-NMR results revealed that steam had little effect on the hydrothermal stability of Mg-saponite but had significant effects on saponites which have Zn^2+^ incorporated in the octahedral sheets. Stability of saponites incorporating Zn^2+^ decreased. For example, saponites where Mg/Zn is 2 in the octahedral sheets showed a decrease in crystallinity; Al atoms within the saponite structure were also observed to move out to non-framework positions [44]. 

### 3.3. Thermal Stability in H_2_

The thermal stability of selected saponites (Ni^2+^-, Co^2+^-, and Cu^2+^-containing saponites, all exchanged with Al^3+^) synthesized by Vogels et al. [44] were also studied under reducing conditions. Thermal treatment was conducted as follows. Dry samples typically weighing around 100 mg were placed in a quartz reactor through which passed a 50 mL/min gas flow of a 10% v/v H_2_/Ar mixture. The temperature was then increased from 25 to 800 °C at a rate of 5 °C/min. A CO_2_ (s/g) cold trap was used to freeze out water formed during dehydration of the sample and during reduction. The amount of H_2_ consumed was measured with a hot wire detector placed beyond the cold trap. The method outlined here is termed as temperature programmed reduction (TPR).

**Figure 12:**
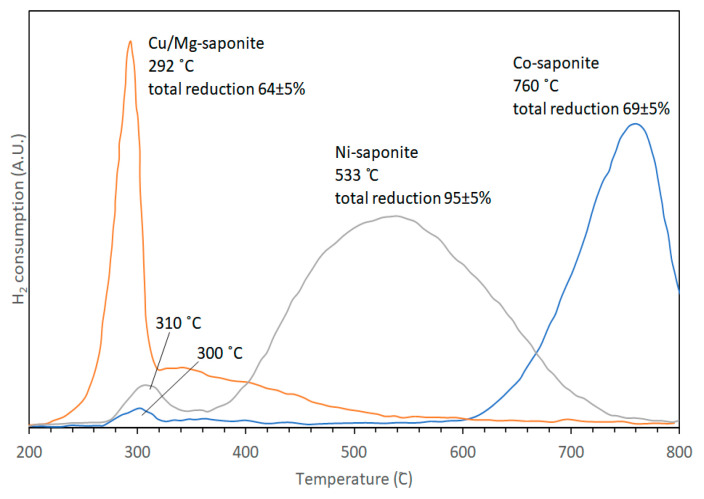
Temperature-programmed reduction profiles of Cu-, Ni-, and Co-containing saponites. Modified from Figure 6 in [44].

Results of TPR experiments showed that saponites with octahedral sheets consisting of pure Mg^2+^ and/or Zn^2+^ were not reduced within the temperature range measured (25–800 °C). Cu^2+^-containing saponites, however, are most easily reduced, beginning at 150 °C with the rate of reduction increasing sharply to a peak maximum at 292 °C followed by a broad band at higher temperatures (Figure 12). The crystallinity of synthetic Cu-containing saponite as determined in [27] was poor and was suggested to be the cause of its easier reduction. Compared with the reduction temperatures observed for chrysocolla [Cu_2_Si_2_O_5_(OH)_2_] [91,92], the reduction of the synthetic Cu^2+^-containing saponite proceeded at higher temperatures. This was attributed to better accessibility of the octahedral sheets of chrysocolla by hydrogen, being covered only on one side by a tetrahedral sheet. In contrast, the octahedral sheets of Cu^2+^-containing saponites are covered on two sides by tetrahedral sheets. Ni- and Co-saponite were more resistant to reduction than Cu-saponite. The TPR profile of Ni-saponite showed two reduction steps: a small peak centered around 310 °C and a large broad peak (FWHM ~ 200 °C) centered at 533 °C. Although not shown by XRD, the first TPR peak is similar to the reduction peak of Ni(OH)_2_ [93]. It was possible that some formation of Ni(OH)_2_ occurred during the authors’ synthesis of Ni-saponite. The second large reduction step was attributed to the reduction of Ni^2+^ present in the saponite structure as the shape was comparable albeit at lower temperatures (by 50 to 150 °C) to the reduction profiles obtained with nickel hydrosilicates as reported by Brahma in 1990 and Van de Loosdrecht in 1995 as cited by Vogels et al. [44]. This observed shift to lower temperatures was rationalized by taking Ni-talcs studied by Carriat et al. [92] as analogy. A decrease in the particle size of Ni-talcs resulted in the shift in reduction maximum towards lower temperatures. This finding strongly points to a reaction rate controlled by phase boundaries. Co-saponite resisted reduction more strongly than Ni^2+^- and Cu^2+-^containing saponites. Similar to Ni-saponite, the TPR profile of Co-saponite exhibited two maxima at 300 and 760 °C. The small peak at 300 °C probably originated from the reduction of Co(OH)_2_. It seems plausible that the formation of some Co(OH)_2_ occurred next to Co-saponite during synthesis, similar to Ni(OH)_2_. The amount of Co(OH)_2_, measured with TPR, never exceeded 3%. The main reduction step was sharper as compared to that of Ni-saponite: reduction started at 600 °C and was not finished at the maximum measurement temperature of 800 °C, resulting in a final degree of reduction of Co-saponite of only 69%. It is remarkable that Co^2+^ in a silicate lattice is far more difficult to reduce than Ni^2+^, even though both (hydr)oxides exhibit similar reducibility behaviors.

## 4. Catalytic Properties

Understanding the catalytic properties of synthetic mineral surfaces in the laboratory would provide more information on how life could have emerged. Several clay minerals including saponites were studied for their potential role in catalyzing reactions in primitive Earth. For example, Ferris and co-workers demonstrated clay-catalyzed synthesis of polynucleotides. RNA oligomers containing 6−14 monomer units were synthesized from 5-phosphorimidazolide of adenine (ImpA) in the presence of montmorillonite [16]. They have also shown that longer chains (~50 mers) of prebiotic molecules can be obtained by incubating the activated monomers with minerals (montmorillonite for nucleotides; illite, and hydroxylapatite for amino acids) [94]. Ferris and his co-workers performed studies on formation of biopolymers near the lower temperature limit. This was to address the sensitivity to hydrolysis of the oligomers and their precursors at higher temperatures. Nevertheless, several proponents support the notion that the origin of life may have started in hydrothermal vents as cited by Martin et al. in their review paper, Hydrothermal vents and the origin of life [12]. Williams showed that smectites (montmorillonite and saponite) may have formed a “primordial womb” where methanol enters, incubates, and synthesizes into organic molecules (to C20) under seafloor hydrothermal conditions [12]. Meñez et al. [17] proposed that Fe-rich saponite could have catalyzed the abiotic synthesis of aromatic amino acids found preserved at depths beyond the Atlantis Massif. Interestingly, the authors proposed that these syntheses may have been possible through Fe-rich saponite-catalyzed Friedel–Crafts-type reactions during the hydrothermal alteration of oceanic peridotites. Such proposal was based on the observed properties of Fe-rich smectites namely pillaring effect, enhanced sorption capacity, high reducible iron content as well as on studies showing that Fe-smectites are the most efficient solid catalyst for Friedel–Crafts reactions [95]. Friedel–Crafts-type reactions are the industry-preferred method for the alkylation of (hetero)arenes under the catalytic effect of Lewis or Brønsted acids. Indeed, in the works of Vogels et al. [27,44,45,50] on urea-assisted, low temperature synthesis of saponites with different octahedral (Mg, Zn, Ni, and Co) and tetrahedral metals (Al, Ga, and B), Friedel–Crafts alkylation of an arene (benzene) is one of the main reactions featured. While the saponites of Vogels et al. [27,44,45,50] were not synthesized to lend credence to the clay-catalyzed synthesis of prebiotic molecules and advance understanding on the origin of life, it is important to note that their works provided information on the saponites’ adsorption properties, CEC, thermal stability, and the effects of tetrahedral and octahedral substitution on Lewis or Brønsted acidity and on catalytic performance. This information will be important for researchers who would want to use the methods of Vogels et al. in preparing saponites that can be used for probing their potential role in catalyzing prebiotic reactions. 

Most of the catalytic reactions studied using the saponites of Vogels et al. were done at harsher temperatures than normally employed in prebiotic synthesis studies. Nevertheless, discussing the reactions in this review might provide insights on the inter-relationships between structure/properties of saponites and their catalytic performance whether in industrially important reactions or in prebiotic reactions.

### 4.1. Cracking of n-Dodecane

In the catalytic cracking of n-dodecane, measurements were performed in a fixed-bed nanoflow pulse system at reaction conditions of 450 °C and 4 bar total pressure. The details are described as follows: The catalyst bed was filled with 20 wt% synthetic saponite and 80 wt% silica, both of which were between 0.212 and 0.300 mm in size. The gas flow composing helium, *n*-dodecane, and nitrogen at concentrations of 19.5, 12.5, and 67.9 mol%, respectively, were pulsed at a weight hourly space velocity (WHSV) of ~66 h^−1^ during the pulses. The n-dodecane/catalyst ratio was set at 0.18 g/g. Gas chromatography was used to analyze the products in which the He tracer gas and H_2_ were detected with a thermal conductivity detector (TCD) followed by the analysis of the hydrocarbons C_1_ up to and including C_5_. A backflush was performed, and the peak was used to determine the number of longer hydrocarbons (C_6_–C_12_). The catalyst activity was assumed to follow the first reaction order reported by Corma, Miguel and Orchillés [96] on zeolite-catalyzed n-alkanes cracking and the rate constant expressed as k:k = [−ln(1 − 0.01(C_5_-yield))]/[ct ∗ cf], where C_5_-yield, ct, and cf are the total amount of C_1_ to C_5_, the contact time, and the catalyst fraction, respectively [50].

**Figure 13:**
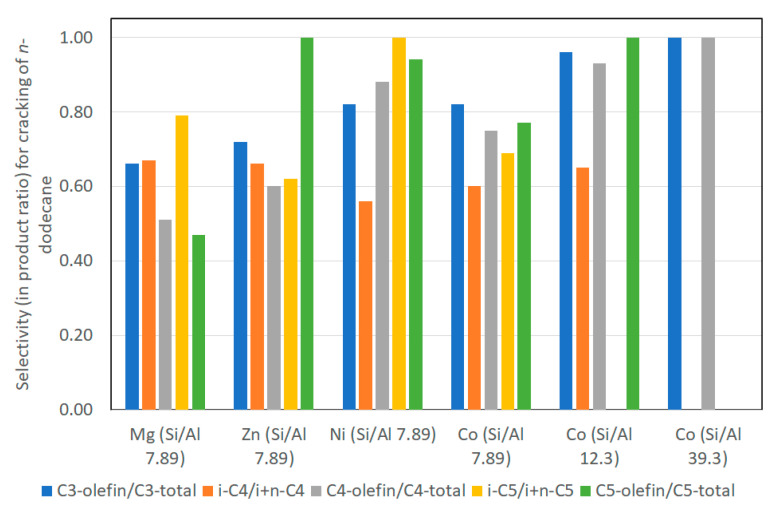
Selectivity expressed in product ratios for the cracking of n-dodecane. C = paraffin, i = iso, n = normal, total = total amount of olefins and paraffins. This figure is based on Table 2 of Vogels et al. [50].

The observed initial first-order rate constant (k) was highest for Mg-saponite followed by Co, Ni, and Zn. These results were explained based on the combined effects of surface area, acid strengths and ease of reduction of the metal cations in the octahedral sheets. The specific surface area of the saponites were reported as follows: 600–750, 500–600, 400–500, and 100–300 m^2^/g for Mg-, Ni-, Co-, and Zn-saponites, respectively. In terms of ease of reduction by hydrogen, neither Mg^2+^ or Zn^2+^ in the octahedral sheets can be easily reduced even when temperatures were raised to 800 °C. Thus, the catalytic cracking behavior of Mg- and Zn-saponites is due mainly to Lewis acid centers. However, carbonaceous deposits on the stronger (Lewis) acid sites resulted in substantial deactivation of Mg-saponite. Ni^2+^ and Co^2+^ ions were more susceptible to reduction by hydrogen and this resulted in saponite structure collapse. This in turn led to a loss or reduction of acid properties and subsequent lower catalytic activity. Nevertheless, the presence of Co and Ni metallic particles in the interlayers of the saponites afforded catalytic bifunctionality (both the metal particles and the acid sites were catalytic) for the Ni- and Co-saponites. The selectivity for C_1_–C_5_ products was remarkable for Ni- and Co-saponites as shown in Figure 13. During the catalytic cracking experiments, carbon deposition occurred on metallic particles (especially on nickel where larger metallic particles were formed) and subsequently reacted with hydrogen to form methane. (Reduced) Co-saponites exhibited a high selectivity for C_3_-, C_4_-, and C_5_-olefins. This can be attributed to the smaller Co particles formed where coke build up cannot proceed rapidly, thus affecting the rate of methane formation as well. As a result, dehydrogenation prevailed in saponites containing Co particles, hence, the high selectivity for C_3_- to C_5_- olefins. The effect of variation in Si/Al ratio on C_1_–C_5_ selectivity was detected only for Co-saponites [50].

### 4.2. Hydro-Isomerization of n-Heptane

In the petroleum industry, isomerization is an important reaction as it converts n-alkanes into their isoparaffins of higher octane number. This paragraph discusses the isomerization reactions of n-heptane performed by Vogels et al. [50] in a catalytic test unit with a fixed bed reactor at a total pressure of 30 bar. Temperatures were decreased and increased between 160 and 400 °C at a rate of 0.22 °C/min during reactions. The feed composition was 1:4 mol/mol ratio of *n*-heptane and hydrogen with the gas hourly space velocity (GHSV) of 1120 mL(stp)/(g h). The products were analyzed with a gas chromatograph. Catalytic activity was reported as the temperature at 40% conversion. All saponite samples used in catalytic studies were pretreated (see details in Vogels et al. [50]) and were H^+^ exchanged.

**Figure 14:**
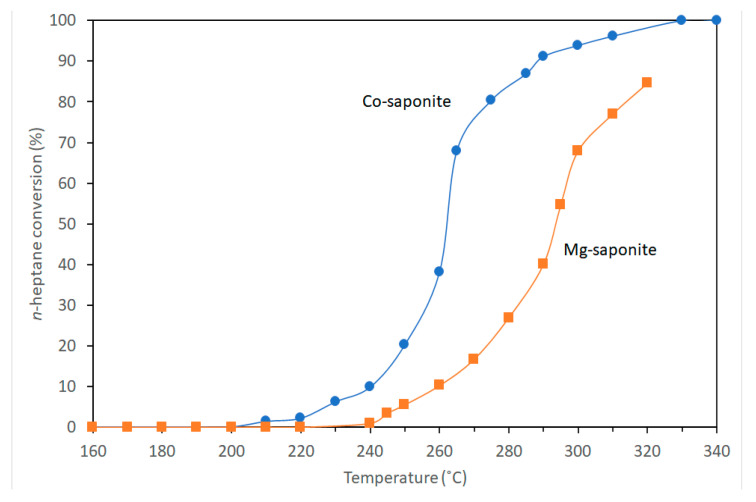
The conversion of *n*-heptane at rising reaction temperatures of Co-saponite (Si/Al ratio 7.89) and Mg-saponite (Si/Al ratio 7.89). Modified from Figure 3 in [50].

The synthetic saponite possessed almost exclusive presence of Lewis acid sites within the H^+^ exchanged clays. As such, it would be reasonable to relate the extent of acid-catalyzed isomerization reactions on the saponites to the Lewis acidity. Results of *n*-heptane conversion as a function of temperature for Co- and Mg-saponite (Figure 14) show significantly higher conversion measured for the Co-saponite than that measured for Mg-saponite with the same Si/Al ratio. The difference in temperature at 40% conversion was 36 °C. Notwithstanding the high activity of Co-saponite, the selectivity for *i*-heptane was low at approximately 15% *i*-C_7_ yield between 40 and 65% conversion. In contrast, the *i*-C_7_ selectivity of Mg-saponite was 85–90%. The high selectivity was retained up to 80% conversion. These differences were attributed to possible reduction of cobalt in the saponite crystal structure thereby leading to cracking instead of isomerization of *n*-heptane on the surface of metallic Co particles. It is interesting to note that the Si/Al ratio of the saponites did not show a consistent effect in the catalytic performance. Mg-saponite showed a slightly higher activity with decreasing Si/Al ratio at decreasing temperatures but opposite behavior in subsequent measurements at increasing temperatures. When Ni or Co were incorporated and the saponites have a mixed-metal octahedral sheet, the reaction profiles from 400 to 200 °C were completely different from the profiles measured with the saponites containing exclusively Ni or Co. The measured activities were considerably lower but the *i*-C_7_ selectivities increased significantly. This was attributed to the accumulation of carbon on the metallic particles rendering Ni or Co particles in the saponites less effective in *n*-heptane cracking and the remaining (Lewis) acid sites more active in catalyzing the isomerization reaction. 

The efficiency of the synthetic saponite in paraffin isomerization reactions is higher compared with an amorphous silica alumina (ASA) catalyst but lower than a commercial HZSM-5 (H^+^ exchanged Zeolite Socony Mobil-5) catalyst. Nevertheless, it is clear that Lewis acid sites of the synthetic saponites are active towards paraffin isomerization reactions. Saponites with high Lewis acid sites may be significant in modelling polymerization and isomerization reactions of early Earth. It is interesting to note that a computational study by Rimola, Ugliengo, and Sodupe [97] on formation versus hydrolysis of the peptide bond on mineral surfaces points to a combined influence of Lewis and Brønsted sites in the minerals and the London forces acting between the biomolecules and the inorganic surface on: (i) condensation of glycine to yield oligopeptides as reaction products; (ii) inhibition of the hydrolysis of the resulting oligopeptides. While completely different and more complex, isomerization processes (preferably one that preserves homochirality) of prebiotic molecules can possibly occur in hydrothermal compartments, where simple molecules can concentrate on mineral surfaces and polymerize [98,99].

### 4.3. Friedel–Crafts Alkylation of Benzene with Propylene to Cumene

As discussed, Meñez et al. [17] proposed that Fe-rich saponite could have catalyzed the Friedel–Crafts-type abiotic synthesis of aromatic amino acids in hydrothermal environments. The catalyzed Friedel–Crafts reaction performed by Vogels et al. [50] discussed in this paragraph was for industrial applications but the narrative here may be useful in understanding the catalytic activity of synthetic saponites in such reactions. The Friedel–Crafts reaction studied was the alkylation of benzene with propylene to cumene (isopropylbenzene) and was carried out in the research laboratory of Engelhard in De Meern, The Netherlands. The synthetic saponites were sieved and fractions between 0.1 and 0.4 mm and in concentrations of 0.2 or 1.5 wt% were used. These were then calcined under a nitrogen flow for 3 h at the desired temperature. Calcination was performed to remove water but a non-calcined and a wet saponite were also used as control samples to still determine whether water affects the catalytic performance. After the calcination, the saponites were dispersed in dry benzene and transferred to a stainless-steel autoclave. Propylene was continuously mixed into the autoclave containing an excess of benzene. The temperature was then increased to the desired level. The excess benzene (experimental benzene/propylene ratios were between 7 and 8) was necessary to avoid multiple alkylation. Details of the benzene/propylene molar ratios, catalyst, reaction temperature, and reaction duration are found in [50]. The resulting products were analyzed on a gas chromatograph with a capillary Chrompack CP-Sil-CB column and the conversion and selectivity were calculated as follows:

(1) $$\mathrm{Conversion}\;=\;\frac{mole\;cumene+2\left(mole\;DIPB\right)+3\left(mole\;TIPB\;formed\right)}{mole\;propylene\;in\;starting\;mixture}\;\times \;100\%$$

(2) $$\mathrm{Selectivity}\;=\;\frac{mole\;cumene\;formed}{mole\;cumene+\;2\left(mole\;DIPB\right)+3\left(mole\;TIPB\;formed\right)}\;\times \;100\%$$

where DIPB and TIPB stands for diisopropylbenzene and triisopropylbenzene, respectively. Products formed with negligible concentration such as *n*-propylbenzene and of oligomers of propylene were not included in the calculations.

**Figure 15:**
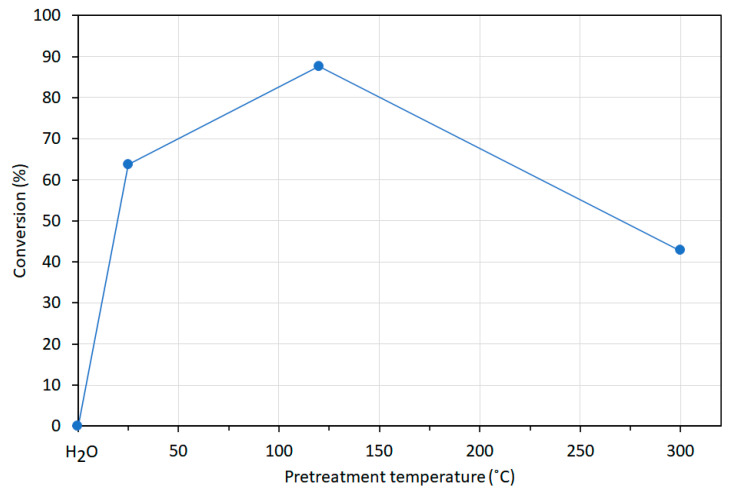
The influence of the hydration state of 0.2 wt% Zn-saponite (Si/Al ratio 7.89) on the catalytic activity. H_2_O means wet saponite sample. Reaction conditions: temperature 160 °C, duration 0.25 h, benzene/propylene molar ratio ~7. Modified from Figure 5 in [50].

The conversion performance of 0.2 wt% Zn-saponite at 120 °C was 87% after 0.25 h (Figure 15). This amount is higher (99%) when using 1.5 wt% Zn-saponite at 160 °C with the selectivity not changing much between 73% and 84%. The presence of water (labeled H_2_O in the origin of the horizontal axis) in the feed resulted in a completely deactivated Zn-saponite. This was attributed to blocking by water of the acid sites rendering the saponites inactive towards the reactants. Increasing the pretreatment temperature to 300 °C resulted in the activity to decrease to ~40%. This catalytic behavior was correlated with the acid properties observed for Zn-saponites with interlayer Al^3+^ where it exhibited a much higher acidity when dried at 150 °C compared when calcined at 350 °C. The dissociation of water at 150 °C within the interlayer of the exchangeable Al^3+^ cations may have formed Brønsted acid sites in the Zn-saponite structure. However, the nature of acid sites was not investigated. This would have been more conclusive had temperature programmed desorption-thermogravimetric (TPD-TG) analyses of n-propylamine adsorbed on saponites were performed and the results coupled with DRIFTS (diffuse reflectance infrared Fourier transform spectroscopy) runs of pyridine adsorption on the catalysts [100,101]. Adsorption of pyridine on Brønsted acid sites are typically at 1515–1565 cm^−1^ in the IR spectra and characteristically different from adsorption on Lewis acid sites typically at 1435–1470 cm^−1^ [101]. TPD-TG, on the other hand, has been used to reveal specific interactions of n-propylamine with Brønsted acid sites on the surface of acidic supports, such as zeolites and silica-aluminas and was shown to quantify the amount of these acid sites better [100,102].

**Figure 16:**
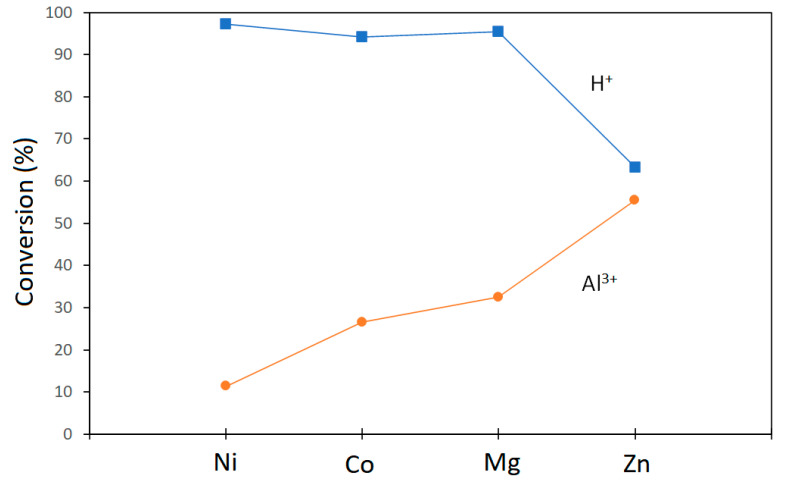
The influence of the composition of the octahedral sheet (Ni^2+^, Co^2+^, Mg^2+^, and Zn^2+^) with (blue) H^+^- and (orange) Al^3+^-exchanged saponites (Si/Al ratio 7.89) on the catalytic performance. H^+^-saponites: 1.5 wt% catalyst at 190 °C and 2 h, Al^3+^-saponites: 0.2 wt% catalyst at 160 °C for 0.25 h. Modified from Figure 6 in [50].

The effect of the type of octahedral and the interlayer cation on the catalytic performance of Al^3+^- and H^+^-exchanged saponites was also investigated and the results shown in Figure 16. The H^+^-exchanged Ni-, Mg-, and Co-saponites show high catalytic activity with conversion reaching around 95% while that of Zn-saponite was substantially lower (63%). Al^3+^-exchanged saponites, on the other hand show an opposite trend, recording low conversion for Ni-, Mg-, and Co-saponites but higher conversion with Zn-saponite. The trend in H^+^-exchanged saponites was rationalized based on the surface area. The interlayer space of these saponites might be too small to permit catalytic reactions. This would be more pronounced when the proton has transferred to the tetrahedral sheet cavities thereby limiting catalytic reactions to the external surface of the clay particles. The specific surface area of Zn-saponite was significantly lower than those of the Mg-, Ni-, and Co-saponites [27] which could have resulted in a lower catalytic activity. More isomorphous substitution of Si^4+^ by Al^3+^ increased the catalytic activity of H^+^-exchanged Zn-saponite possibly due to an increase in the number of acid sites on the external surfaces. Low amount of Al^3+^ in the tetrahedral sheets of Al^3+^-exchanged saponites resulted in a low number of acid sites, but with a relatively strong acidity. The selectivity of the Zn-saponites were also determined. An Al^3+^-exchanged Zn-saponite showed a strong selectivity for *p*-DIPB. The *o*-DIPB isomer was also formed but the *m*-DIPB was present in much lower quantities even though it is the more thermodynamically favored isomer (Table 2). The authors noted that this is even more apparent at higher reaction temperatures and longer duration when *p*- and *o*-DIPB are formed almost exclusively. Dealuminated zeolites also showed similar shape selectivity where *p*-DIPB was the preferred product [103,104]. Unlike in saponites, however, the ortho isomer was hardly formed in dealuminated zeolites. Shape selectivity was also reported in the pillared saponite-catalyzed alkylation of toluene by methanol to form xylene where the para isomer was formed in quantities greater than thermodynamic equilibrium calculations [105]. All of these studies attributed shape selectivity to the characteristic texture and porosity of the catalysts [50,103,104]. When micropores exist in these catalysts, shape-selective sorption and molecular sieving sometimes arise. These micropores can be affected by varying the size and shape of interlayer cations as well as by the charge and charge density of the interlayers. It would be interesting to see whether this shape selectivity of saponites apply to other reactions especially in the formation of biomolecules or their simple precursors and whether tuning the interplay of factors affecting shape selectivity can drive the exclusive formation of one isomer.

## 5. Summary

Saponite clays are easy to synthesize in large and pure form, have tunable properties, and are shown to catalyze organic reactions. The method involving urea is presented as a reasonable analog of natural processes. The aluminosilicate gels in the first step of the synthesis form a 4-fold-coordinated Al^3+^ similar to what is found in nature, such as in volcanic glass. The use of urea, a compound figuring in many prebiotic model reactions, circumvents the formation of brucite in the final saponite product by slowly releasing ammonia. This controls the hydrolysis of magnesium and enables the formation of saponite without the accompanying sudden increase in pH which would have precipitated brucite. The facile formation of the synthetic saponite is easily followed and verified by analytical tools such as XRD, vibrational spectroscopies, XRF, MAS-NMR, EXAFS, and TEM. These tools also prove valuable in probing the integrity and the changes in the local environment of the saponites as they undergo thermal treatment whether in the presence of water or reducing environment or when they promote organic reactions on their surfaces. While the organic reactions catalyzed by saponites in this review are not the reactions that would lend credence to studies on clays and the origin of life, these studies suggest clay surfaces may have provided sites where prebiotic molecules adsorb and undergo more complex reactions. 

## Figures and Tables

**Table 2:** Selectivity towards *p*-, *o*-, and *m*-diisopropylbenzene (DIPB) for Al^3+^-exchanged Zn-saponite (Si/Al ratio = 39.0, dried at 120 °C).

Conditions (Catalysts wt%, Reaction Temperature and Reaction Time)	Conversion (%)	*p*-DIPB (%)	*o*-DIPB (%)	*m*-DIPB (%)
1.5 wt% catalyst, 190 °C, 2 h	99	47	50	3
1.5 wt% catalyst, 160 °C, 0.25 h	98	47	36	17
0.2 wt% catalyst, 160 °C, 0.25 h	87	49	35	16
